# Blood glutamate scavengers increase pro-apoptotic signaling and reduce metastatic melanoma growth in-vivo

**DOI:** 10.1038/s41598-021-94183-8

**Published:** 2021-07-19

**Authors:** Yona Goldshmit, Rita Perelroizen, Alex Yakovchuk, Evgeni Banyas, Lior Mayo, Sari David, Amit Benbenishty, Pablo Blinder, Moshe Shalom, Angela Ruban

**Affiliations:** 1 Steyer School of Health Professions, Sackler Faculty of Medicine, Tel-Aviv University, P.O. Box 39040, 6997801 Tel Aviv, Israel; 2 Australian Regenerative Medicine Institute, Monash Biotechnology, 15 Innovation Walk, Clayton, VIC 3800 Australia; 3 Shmunis School of Biomedicine and Cancer Research, George S. Wise Faculty of Life Sciences, Tel Aviv University, 6997801 Tel Aviv, Israel; 4 Sagol School of Neuroscience, Tel Aviv University, 6997801 Tel Aviv, Israel; 5 Department of Biological Regulation, Weizmann Institute of Science, 7610001 Rehovot, Israel; 6 Neurobiology Department, George S. Wise Faculty of Life Sciences, Tel Aviv University, 6997801 Tel Aviv, Israel

**Keywords:** Cancer therapy, Metastasis

## Abstract

Inhibition of extracellular glutamate (Glu) release decreases proliferation and invasion, induces apoptosis, and inhibits melanoma metastatic abilities. Previous studies have shown that Blood-glutamate scavenging (BGS), a novel treatment approach, has been found to be beneficial in attenuating glioblastoma progression by reducing brain Glu levels. Therefore, in this study we evaluated the ability of BGS treatment to inhibit brain metastatic melanoma progression in-vivo. RET melanoma cells were implanted in C56BL/6J mice to induce brain melanoma tumors followed by treatment with BGS or vehicle administered for fourteen days. Bioluminescent imaging was conducted to evaluate tumor growth, and plasma/CSF Glu levels were monitored throughout. Immunofluorescence staining of Ki67 and 53BP1 was used to analyze tumor cell proliferation and DNA double-strand breaks. In addition, we analyzed CD8, CD68, CD206, p-STAT1 and iNOS expression to evaluate alterations in tumor micro-environment and anti-tumor immune response due to treatment. Our results show that BGS treatment reduces CSF Glu concentration and consequently melanoma growth in-vivo by decreasing tumor cell proliferation and increasing pro-apoptotic signaling in C56BL/6J mice. Furthermore, BGS treatment supported CD8^+^ cell recruitment and CD68^+^ macrophage invasion. These findings suggest that BGS can be of potential therapeutic relevance in the treatment of metastatic melanoma.

## Introduction

It has been known for some time that excitatory neurotransmitter Glutamate (Glu) plays a fundamental role in excitatory neurotransmission in the brain, and has become the focus of many research endeavours since. This research includes the contribution of Glu to the progression of brain cancers, and suggests that Glu is involved in the ability of brain tumor cells to grow, invade, and destroy brain tissue^1–5^. In addition, it has been shown that most brain and peripheral cancer cells secrete Glu mainly via a glutamate/cystine (xc−) antiporter^6,7^. The xc− antiporter functions primarily in the production of the antioxidant glutathione, by exchanging an intracellular Glu molecule for an extracellular cystine molecule, a precursor in glutathione synthesis^7^. Even more so, blocking Glu receptor activation, directly via receptor antagonists or indirectly via blocking Glu secretion, has been shown to increase apoptosis in tumor cells, as well as decrease their proliferative, migratory, invasive, and metastatic abilities^1,5,8,9^. The abundance of research suggesting that extracellular Glu accumulation is involved in glioblastoma progression has prompted us to hypothesize the existence of similar mechanisms in other brain tumors, such as brain melanoma metastases. Supporting this hypothesis, it has been shown that the presence of ectopic metabotropic glutamate receptor 1 (mGluR1) in murine melanocytes results in the formation of melanoma. Additionally, mGluR1 signaling inhibition results in apoptosis of human melanoma cells via cell cycle arrest, as shown in both in vitro and in vivo studies^8,10,11^. When an mGluR1 antagonist or Riluzole, a glutamate release inhibitor, was used to treat melanoma cells expressing mGluR1, cell proliferation, tumor growth, and extracellular glutamate levels were all decreased significantly, with tumor growth decreasing by up to 50%^8,9,11^. Moreover, 100% of transgenic mice with conditional mGluR1 expression in melanocytes have been reported to produce melanomas within 52 weeks following transgene activation^12^. Melanoma mGluR1 hyperactivation is known to act independently of the BRAF and NRAS protooncogenes^13^. Inhibition of melanoma growth in melanoma-bearing mice has also been shown through subsequent inactivation of the mGluR1 transgene. This is in contrast to the progression of melanoma in mice with untampered mGluR1 expression^12,14^. Additionally, it has been demonstrated that transformed melanocytes release high levels of Glu^15^. Taken together, the literature suggests that mGluR1 expression is linked with increased extracellular Glu and melanoma cell proliferation, while its inhibition is linked to their decrease.

Approximately 50% of patients with metastatic melanomas develop brain metastases during the course of their illness^16^. Patients who are diagnosed with advanced malignant melanoma have a 5-year survival rate of about 5%. While the median survival (MS) of conventionally-treated patients with brain metastasis is below 12 months^17^, novel checkpoint therapy (e.g., anti-CTLA-4 antibody and anti-PD-1 antibody), had prolonged MS to 19.2 months, 37.9 months and 12.7 months, respectively^18^. Additionally, it is possible that more advanced tumors with stage III and IV metastases have a higher frequency of mGluR1 expression and of ectopic mGluR1^19–21^. In such cases, the reduction of extracellular brain glutamate could be effective in the management of brain metastatic melanomas.

Recent combined therapies that enhance the immune system are encouraging, however they are limited due to the relative immune resistance that melanoma cells can develop^22–25^. At present, the success of immunotherapy for primary and secondary brain tumors appears to depend on enhancing tumor-specific CD8^+^ T cell immunity since CD8^+^ T cells are strongly associated with direct tumor killing, and thus patient survival^26–28^. As such, developing therapeutic modalities that promote CD8^+^ T cell responses is a key goal in the field of anti-cancer immunotherapy. It has been shown that a low concentration of Glu can promote T cell proliferation and migration, while a high concentration can inhibit it^29–31^. This unique ability of Glu, to induce divergent effects on activated T cells in a concentration-dependent manner, may be important in ongoing anti-tumor immune responses.

Previous failures to control Glu excitotoxicity in humans, due in part to adverse side effects related to the disruption of glutamate necessary for normal cellular signaling, have prompted us to re-evaluate the current strategies. We hypothesized that excess interstitial/cerebrospinal fluid (ISF/CSF) Glu can be eliminated by increasing the rate of the naturally-occurring brain-to-blood Glu efflux^32–34^. We further surmised that such an increase could result from lowering plasma Glu levels, thus increasing the driving force required for Glu flux from brain ISF/CSF to blood. We have previously demonstrated this technique in paraoxon intoxication, ischemic stroke and spinal cord injury models^35–37^. Indeed, the blood glutamate scavenging (BGS) approach has been found to be beneficial in attenuating the progression of glioblastomas by reducing brain Glu levels^5^. Supplementing recombinant human GOT1 (rGOT1) and its co-substrate, oxaloacetic acid (OxAc), in the blood causes a rapid decrease in blood Glu levels, leading to a larger Glu gradient between the brain and the blood that promotes excess extracellular Glu efflux^35–37^. The reported data indicates that such a BGS treatment for reducing brain metastatic growth can be a promising new path in the treatment and management of metastatic cancer.

## Materials and methods

All experiments were conducted according to the Guidelines for the Use of Experimental Animals of the European Community and approved by the Animal Care Committee of Tel Aviv University.

All in vivo procedures were carried in accordance with the ARRIVE guidelines.

### Melanoma cell culture

The cells studied included three melanoma cell lines: HT144 human metastatic melanoma cell line, Mel 624, metastatic melanoma derivative cells from a patient at the surgical branch of the National Institutes of Health (NIH), and the RET mouse melanoma cell line. These cell lines were chosen based on their mGluR1 expression and metastatic origin. In addition, we chose the RET cell line since it has been shown that the *RET* oncogene is commonly mutated in human melanomas, particularly in desmoplastic melanomas which have an increased risk for brain metastasis. The human metastatic HT144 cell line was purchased from the American Type Culture Collection (ATCC), and the RET cells were kindly donated by Dr. Carmit Levi, Tel-Aviv University. Human astrocytes (ScienCell Research Laboratories (Cat No 1800)) served as a control for basal mGluR1 expression levels in intact brain cells compared to the human metastatic melanoma derivative cells and RET melanoma cell line. The RET cells were double labeled with mCherry and Luc2 (pLNT/Sffv-MCS/ccdB plasmid), sorted for red fluorescence, and selected in culture before intracranial (i.c.) injection as detailed below. RET melanoma cells (5 × 10^3^/2 μl) were implanted in C56BL/6J mice to induce tumors along with their accompanying neovascularization. The cells were cultured in RPMI-1640 supplemented with 10% FBS, Pen-Strep, and 4 mg ml^−1^ glucose at 37 °C under a humidified atmosphere of 5% CO_2_–95% air. Before injection, confluent monolayers of cells were released from the tissue culture flask using 0.25% trypsin, rinsed twice with serum-free RPMI-1640, centrifuged, and washed with sterile BPS.

### Intracranial inoculation of RET cells and BGS treatment protocol

C57BL/6 mice were anesthetized with xylazine (10 mg/kg)/ketamine (70 mg/kg). After disinfection and incision of the skin, 2 μl of RET tumor cells (5 × 10^3^/2 μl) were stereotaxically implanted at the following location: 0.5 mm forward from the bregma, 2.1 mm lateral, and 3.0 mm ventral from the dura as we previously described^38^. RET-implanted mice were randomly divided into two groups following tumor cell inoculation.

Group 1 was the control group. Beginning at 1 h post tumor implantation, subcutaneous administration of 200 μl saline was performed daily. The mice were supplied with tap water for drinking containing 0.3 M NaCl until the termination of the experiment.

Group 2 was the treatment group, and the mice in this group received a priming dose of 2.00 mg/kg rGOT1 in 200 μl saline, administered subcutaneously, 2 h post implantation. This was followed by maintenance doses of 0.25 mg/kg rGOT1 in 200 μl saline performed daily. The mice were supplied with tap water for drinking which contained 0.2 M OxAc whose pH = 6.5 until the termination of the experiment, as has been previously described^38^.

### Bioluminescence analysis of the tumor size

In vivo bioluminescent imaging using an IVIS Spectrum CT (Perkin Elmer) was performed in anesthetized mice injected with RET-mCherry-Luc2 cells. Imaging sessions were conducted at 2-, 7-, and 14-days following tumor cell implantation. Each imaging session lasted between 10 and 20 min following d-Luciferin sodium salt injection (30 mg/ml, 100 ml, i.p; Regis Technologies), as this time frame exhibited a steady and maximal intensity. The analysis was performed using Living Image software (version 4.3.1).

### Glutamate levels in plasma

Venous blood samples were collected from mice in order to confirm BGS peripheral activity. Blood was collected from a submandibular vein into tubes containing Heparin. The tubes were then centrifuged at 13,000 rpm for 5 min, followed by separating the plasma and freezing the tubes immediately at − 80 °C. Glu blood levels were analyzed by reversed-phase HPLC as described below.

### Glutamate plasma and CSF determination assay

We sampled CSF in order to evaluate the impact of tumor growth on brain tissue Glu levels and of rGOT1 on excess Glu. For this experiment, we sampled CSF and blood 3 and 7 days after melanoma cell implantation in BGS and vehicle-control treated mice. The sham control group was added at the 3-day time-point. Animals were anesthetized with a mixture of ketamine/xylazine (ketamine 100 mg/kg, xylazine 10 mg/kg) and CSF samples were extracted from the cisterna magna. Briefly, following mice anesthetization, each mouse’s head was secured with a stereotactic head adaptor, the cisterna magna was exposed, and the mouse’s body was positioned down so that the head formed a nearly 135° angle with the body. CSF was immediately collected from the cisterna magna using a glass capillary tube^37^ and the samples were then frozen on dry ice and stored at − 80 °C [sham (n = 5); vehicle-control (n = 5); rGOT1/OxAc treatment (n = 5)]. Glu levels were analyzed with pre-column derivatization with OPA reagent and separated by reversed-phase HPLC with a scanning fluorescence detector. The excitation and emission wavelengths used were 350 and 460 nm, respectively. Chromatography was performed using the UltiMate 3000 LC system (Thermo Scientific). The amino acid standard mix (Sigma-Aldrich) and samples were precolumn derivatized with OPA reagent solution. The derivatization reagent was 5.0 mg OPA dissolved in 100 µL of methanol and diluted with 900 µL of 0.4 M borate buffer (pH 9.5) and 5 µL of b-mercaptoethanol. Derivatization of amino acids: the standard or CSF sample was mixed with 5 µl of OPA derivatization reagent in the auto-sampler. All of the chromatographic separations were performed on a Thermo Hypersyl Gold 5U column (4.6 mm × 250 mm, 2.5 µl). The amino acid concentration was determined using the peak area and the external standard method.

### Lysate preparation and immunoblot analysis

Cells were plated on a 10 cm culture dish at 1 × 10^6^ cells in RPMI-1640 supplemented with 10% FBS, Pen-Strep, and 4 mg ml^−1^ glucose for 24 h before being lysed. For immunoblot analysis, equal amounts of protein from each sample were loaded and resolved by SDS–polyacrylamide gel electrophoresis through 7.5–10% gels as we have previously described^39^. The gels were electrophoretically transferred to a nitrocellulose membrane. Membranes were blocked, blotted with rabbit anti-mGluR1 (1:1000; Cell Signaling) and mouse anti-actin (1:10,000; MP Biomedical) primary antibodies followed by a secondary antibody linked to horseradish peroxidase. Immunoreactive bands were detected by a chemiluminescence reaction, and protein levels were quantified by densitometric analysis of protein bands using the ImageJ software.

For protein level analysis in-vivo, control/BGS treated mice (n = 5 animals for each group) were perfused with ice-cold PBS 7 days after tumor cells implantation, and brains were removed. Only the tumor area with some surrounding brain tissue was homogenized by a manual homogenizer (Fisher Scientific Med 11-850-56, USA) in a lysis buffer. For immunoblot analysis, equal amounts of protein from each sample were loaded and resolved by SDS–polyacrylamide gel electrophoresis through 7.5–10% gels as described above. The protein levels were quantified by densitometry analysis of protein bands using the ImageJ software. p-STAT1 was compared to total STAT1 of stripped membrane, and Iba1 was compared to actin levels of stripped membrane. Primary antibodies were rabbit anti-p-STAT1 (Ser727) (1:1000; Abcam), mouse anti STAT1 (1:1000, abcam), rabbit anti-Iba1 (1:1000; abcam), and mouse anti-actin (1:10,000; MP Biomedical).

### Immunohistochemistry

Cryostat coronal sections (20 µm) of fixed frozen brains including the tumor area were stained using standard immunohistochemistry. Primary antibodies included rabbit anti-Ki67 (1:400, Thermo Scientific), rabbit anti-cleaved caspase 3 (1:100, Abcam), rabbit anti 53BP1 (1:100, Abcam), rat anti-CD8 (1:250, Abcam), rabbit anti-p-STAT1 (1:400, Abcam), rabbit anti-iNOS (1:20; Thermo Scientific), rat anti-CD68 (1:500; Thermo Scientific), rat anti CD206 (1:500; Bio-Rad), and rabbit anti-IBA1 (1:400; Abcam). Secondary antibodies included Goat anti-rabbit and rat Alexa Fluor 488 and 647 (1:1000, Invitrogen). Nuclei were visualized with DAPI (Sigma). Sections were imaged by fluorescence microscopy using an Axioplan Z1 (Zeiss) epifluorescence microscope. Photomicrographs (1300 × 1030 dpi) were obtained with 25× and 5× Plan-Neofluar (Zeiss, Germany) objectives, and acquired using an AxioCam (Zeiss, Germany) digital camera using AxioVision software (v. 4.4; Zeiss, Germany). Fluorescence density was analyzed using Image J. Images were sized using Adobe Photoshop 11.

A series of 20 μm-thick sections were prepared. We determined the density of the various markers that were examined (Ki67, 53BPI, active caspase 3, CD8, p-STAT1, CD68, iNOS, CD206 and IBA1) by measuring the intensity of the immunofluorescence of each marker in areas of 100 × 100 μm inside of the tumor borders. DAPI labelling and mCherry fluorescence were used to identify the tumor area. Immunofluorescence was measured from at least 50 images randomly taken from the tumor areas using an 200× magnification. All measurements were performed using Image J^37^.

### Flow cytometry and FACS sorting

Mononuclear cells were isolated from the brains as previously reported [Mayo 2014]. Mice were euthanized and subjected to perfusion through the left ventricle with ice-cold sterile PBS. Brains were then removed, minced by gentle MACS™ Dissociator (Miltenyi Biotec), and enzymatically dissociated with 0.05% (w/v) collagenase type III, 0.5% Dispase II, 40 mg/ml DNAse I, 20 mM HEPES in HBSS for 30 min at 37 °C to make a suspension of single cells. Enzymes were inactivated with 20 ml of Ca^2+^/Mg^2+^-free HBSS containing 2 mM EDTA. The digested tissue was triturated and passed through a 100-µM cell strainer. Cells were centrifuged and resuspended in 25% isotonic Percoll, underlined by 75% isotonic Percoll, and centrifuged at 1000×*g* at 20 °C for 30 min. Cells were collected from the 75%–25% interphase, and sorted by FACS (SONY SH800) Isolated cells were stained with fluorophore-conjugated antibodies for CD8 (53–6.7, Biolegend) and CD3 (17A2, Biolegend) for 45 min at 4 °C in the dark.

### Statistical analysis

The Shapiro–Wilk test was applied to examine the distribution of the variables, and our results indicated a normal distribution of all variables except for tumor growth at Day 14. Accordingly, comparisons of the normally distributed variables were conducted using unpaired two-tailed Student’s t-tests using GraphPad Prism 4 software and Mann–Whitney *U* tests using SPSS software version 25 (*P < 0.05; **P < 0.01; and ***P < 0.001). Results are presented as mean ± SD. The tumor volume at Day 14 in the treated vs. untreated group is presented as the median ± interquartile range.

## Results

### mGluR1 expression in murine and humane metastatic melanoma cells

**Figure 1:**
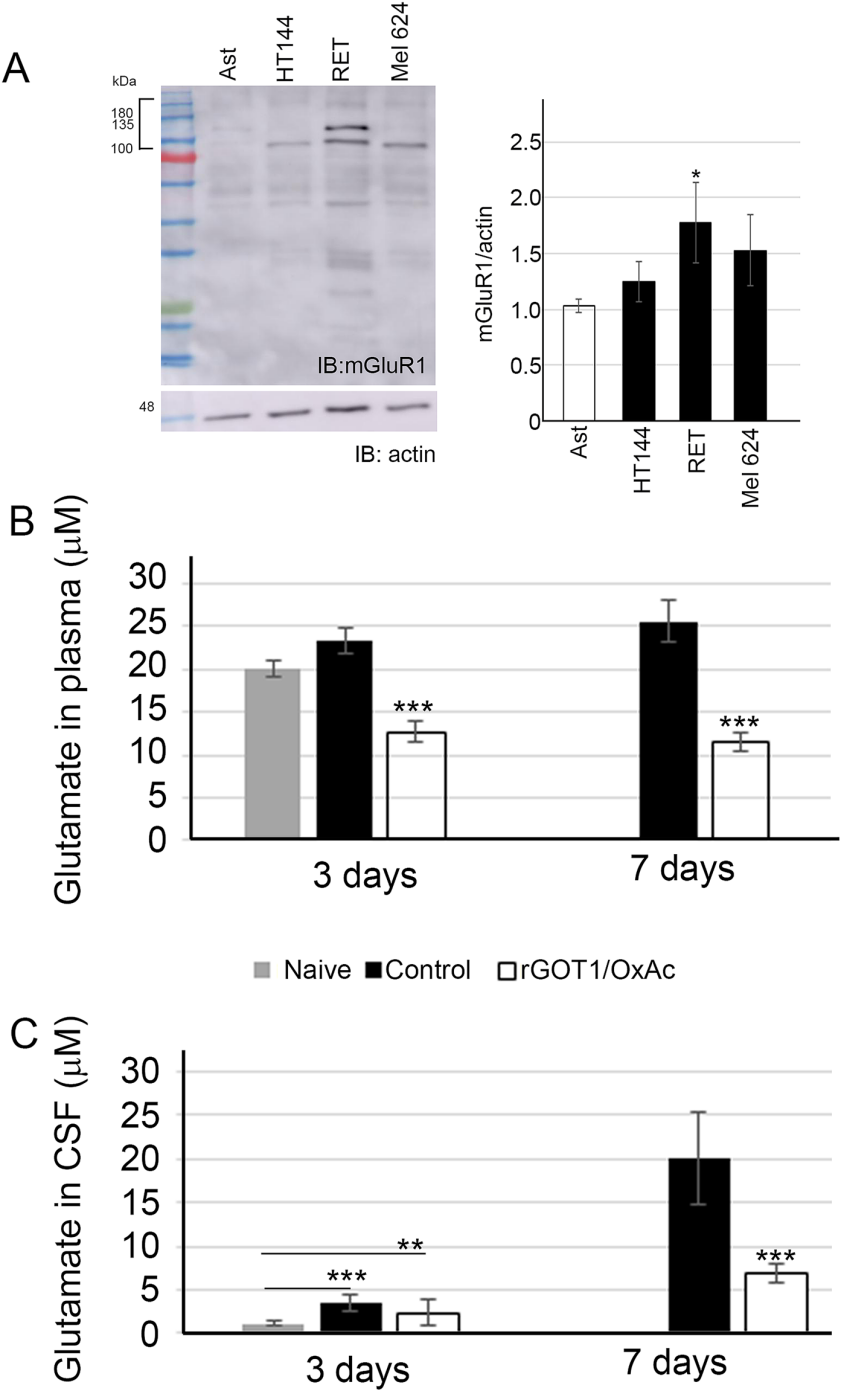
Reduced glutamate levels in the blood and CSF followed by BGS treatment. (**A**) Western blot analysis of mGluR1 expression in different melanoma cell lines. The human astrocyte line was used as a control for a cell line that expresses high levels of mGluR1; the HT144 human melanoma cell line; the RET mouse melanoma cell line; and Mel 624 is patients’ derivative cells from the surgical branch of the NIH. Total cell lysates were immunoblotted with mGluR1 and anti-actin antibody in order to determine the fold induction of mGuR1 levels in melanoma cell lines compared to astrocytes (used as a control). Results are presented as mean ± SD (n = 3 experiments; *p < 0.05). (**B**) Levels of glutamate in the blood of control vs. BGS treated mice i.c. injected with 2 μl of RET melanoma cells (5 × 10^3^/2 μl). Blood samples at day 3 and 7 after tumor cell implantation (n = 5 animals/time-point; n = 5 naive mice). (**C**) Glutamate levels in the CSF of control vs. BGS treated mice i.c. injected with RET melanoma cells. CSF samples at day 3 and 7 after the tumor cell implantation (n = 5 animals/time-point). Results are presented as mean ± SD (n = 5 animals/group; n = 5 naive mice; **p < 0.01, ***p < 0.001).

Previous studies have demonstrated a significant inhibition in melanoma cell growth in vitro and in vivo as a result of blocking the mGluR1 receptor. We first examined mGluR1 expression in various human melanoma cell lines and compared them with the RET mouse melanoma cell line used in this study. The human astrocytes line served as a control for the basal expression of mGlur1 in the brain. Our results demonstrated a significantly higher expression of mGluR1 in HT144 and RET melanoma cell lines, and in one patient-derived melanoma cells compared to its expression in the human astrocyte cell line (Fig. 1A). The highest expression of mGluR1 was detected in RET cells. Since the RET murine melanoma cell line highly expresses mGluR1, this line was utilized for the in vivo experiments.

### BGS treatment decreased glutamate levels in the plasma and CSF in melanoma-bearing mice

To confirm whether BGS would reduce the levels of extracellular Glu due to RET melanoma cell secretion, we examined CSF and plasma Glu concentrations in treated vs. untreated mice. Tumor growth was confirmed by bioluminescence prior to CSF and blood sampling. Our results demonstrate that plasma Glu levels were slightly and non-significantly elevated at Days 3 and 7 in melanoma-implanted mice, compared to naïve mice (Fig. 1B). Furthermore, we verified a significant reduction in plasma Glu levels on Days 3 and 7 post melanoma implantation in the BGS treated group (Fig. 1B), compared to the naive mice or to vehicle-treated mice implanted with RET melanoma cells (naïve mice: 19.98 ± 0.9; vehicle-control group: day 3 23.25 ± 1.5, day 7 25.62 ± 2.5; BGS group: day 3 12.71 ± 1.3 μM, day 7 11.55 ± 1.6 μM, p˂0.001).

In the vehicle-treated mice, CSF Glu levels were significantly elevated at Days 3 and 7 post melanoma cells implantation compared to the naïve mice (Fig. 1C). Moreover, Glu concentration was elevated more than fivefold at Day 7 compared to Day 3 post-implantation (3 days, 3.6 ± 0.87; 7 days, 20.02 ± 5.6, p˂0.001). Three days of treatment with BGS resulted in a non-significant decrease in CSF Glu levels, however, CSF Glu concentration was significantly decreased compared to the vehicle-control group at Day 7 (3 days 2.4 ± 0.88; 7 days 6.9 ± 2.1, p < 0.001) (Fig. 1C). These results verified the ability of BGS treatment to reduce excess levels of Glu that accumulate in the brain parenchyma due to tumor growth.

### Blood glutamate scavengers reduced melanoma growth in vivo

**Figure 2:**
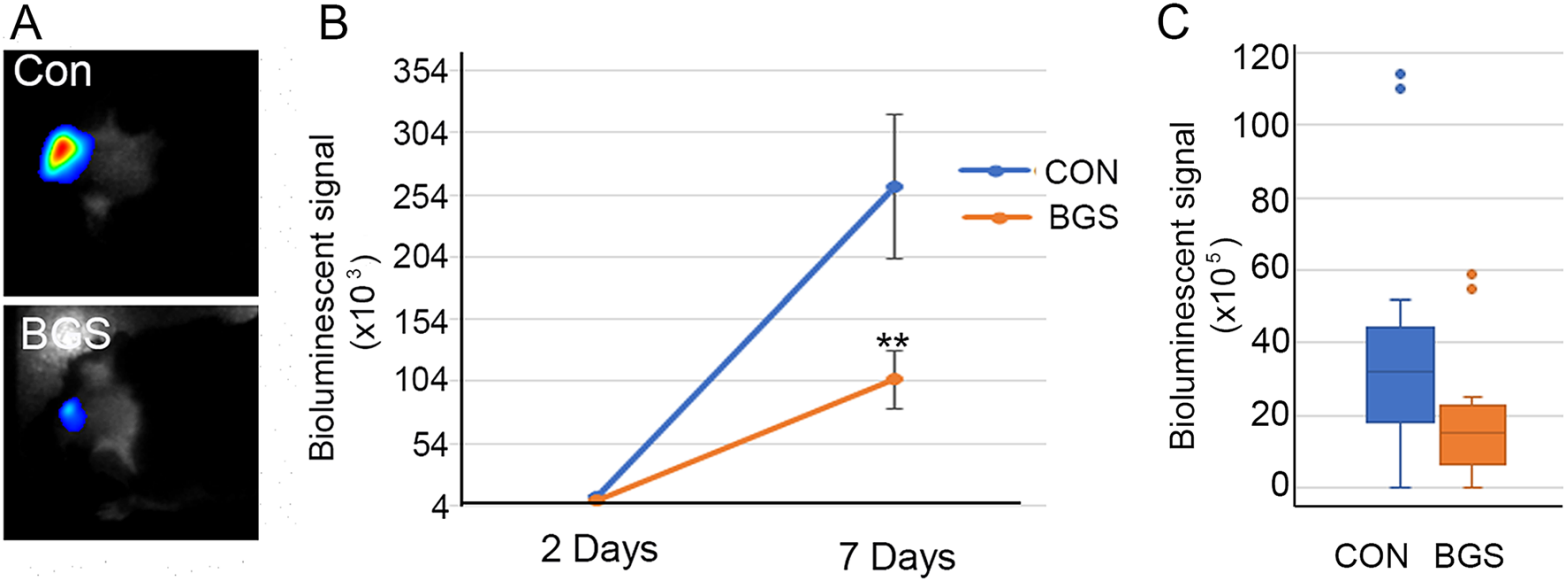
Reduction in tumor size in BGS treated mice. Animals were injected with Luc2-mCherry-labeled RET melanoma cells (5 × 10^3^/2 μl) and examined by bioluminescence imaging at day 2, 7 and 14 after the i.c. cell implantation. (**A**) A representative image of 7 days. (**B**) BGS treated animals show a significant decrease in tumor size at day 7 post implantation compared to the vehicle-control mice. (**C**) BGS treated animals show a trend (not significant) of a decrease in tumor size at day 14 post implantation compared to the vehicle-control mice. Results are expressed as mean ± SD with two-tailed unpaired Student *t* tests for day 2 and 7, and as median ± interquartile range with a Mann–Whitney *U* test for day 14 (n = 10 mice/group). Images on the left demonstrate a representative image of tumor size in BGS treated mice (lower panel) vs. untreated mice (upper panel) for day 7.

In order to examine whether BGS treatment, which can potentially shift Glu secreted by tumor cells from the brain to the blood, affects tumor growth, RET melanoma cells were injected into the right hemisphere. BGS or vehicle treatment began one hour and tumor growth was examined on Days 2, 7 and 14 following tumor implantation. Our results demonstrate that despite the lack of a significant difference between tumor volume in treated vs. untreated mice on Days 2 and 7 of treatment, tumor size was significantly decreased as measured by bioluminescence imaging (control 25.5 × 10^4^, BGS 10.1 × 10^4^ p < 0.01) (Fig. 2A,B). This significant reduction in tumor size in treated animals suggests that the depletion of excess brain Glu by boosting the Glu efflux from the CSF to the blood was sufficient to inhibit melanoma cell growth. On Day 14, there were still differences in tumor size between the groups, however they were not statistically significant (check 43.2 × 105, BGS 19.5 × 105 p = 0.06) (Fig. 2C).

Next, we examined the expression of various proliferation, apoptotic, and inflammatory markers within and around tumor cells in the brain in order to study the effects of BGS treatment at the cellular level. All immunohistochemistry was performed on sectioned brains at Day 7 after implantation and treatments.

### BGS decreased tumor cell proliferation

**Figure 3:**
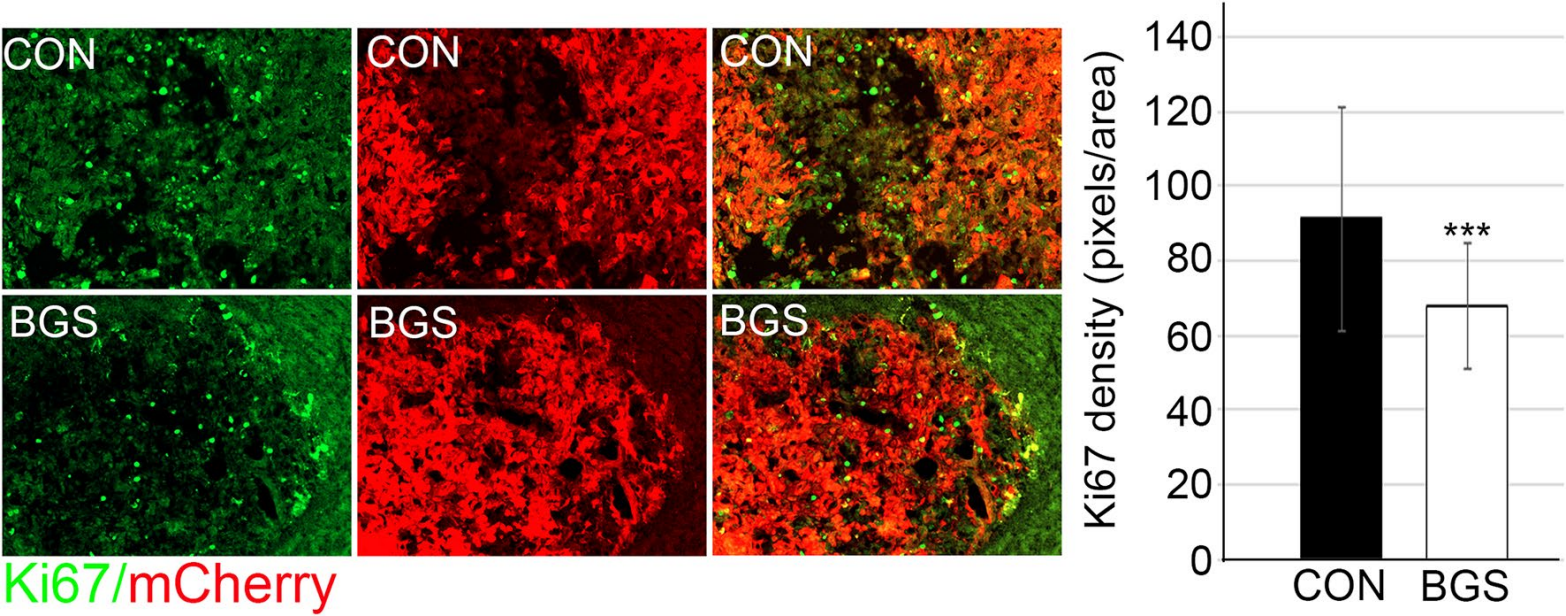
BGS treatment inhibited RET cell proliferation. Representative images of the control and BGS treatments for Ki67 proliferating marker expression (green) in the tumor (mCherry). Ki67 density is significantly reduced in the BGS-treated group compared to the control group. Results are presented as mean ± SD (n = 7 animals/group; ***p < 0.001). Scale bar 200 µm.

We used Ki67 to examine cell proliferation within the tumor mass as it remains one of the most useful adjunct markers to determine malignancy. Numerous studies have shown increased recurrence and mortality rates that directly correlate with increased Ki67^40,41^. Our results confirm that BGS treatment significantly reduced tumor cell proliferation by 25% in treated vs. untreated mice (control 91.1 ± 29.9; BGS 67.8 ± 16.7, p < 0.01) (Fig. 3). These results suggest that one of the effects of BGS treatment is reduced tumor growth due to inhibition of RET cell proliferation.

### BGS increased the expression of a DNA suppressor marker and pro-apoptotic signaling

**Figure 4:**
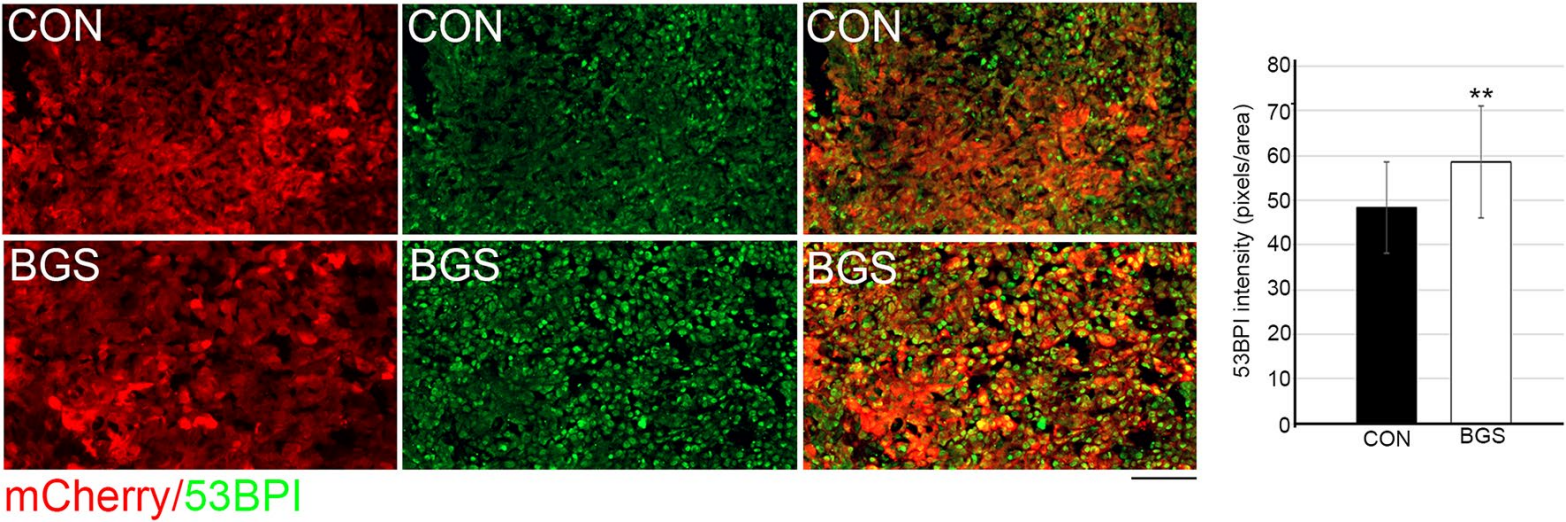
BGS treatment increased RET melanoma cell DNA damage. Representative images of the control and BGS treatments for 53BP1 DNA damage and tumor suppressor marker expression (green) in the tumor (mCherry). 53BP1 density is significantly increased in the BGS-treated group compared to the control group. Results are presented as mean ± SD (n = 7 animals/group; **p < 0.01). Scale bar 200 µm.

Next, we examined various apoptotic markers in order to determine whether BGS, besides for decreasing the tumor proliferation rate, can also promote apoptotic cell death. 53BP1 is a p53 binding protein that binds to the central DNA-binding domain of p53^42^. In response to DNA damage, the protein deposits at the sites of DNA strand breaks and plays a central role in the response of mammalian cells to genotoxic stress^43^. 53BP1-deficient mice are predisposed to tumor development as 53BP1 is essential for an appropriate cellular response to DNA damage in vivo^44^. Therefore, we examined 53BPI expression within tumors in both BGS-treated and control groups. Our results demonstrated a significantly higher expression of 53BPI in the RET cells after 1 week of BGS treatment compared to vehicle-control animals, as measured by fluorescence density (control 48.3 ± 10.3; BGS 58.6 ± 12.5, p < 0.01) (Fig. 4).

**Figure 5:**
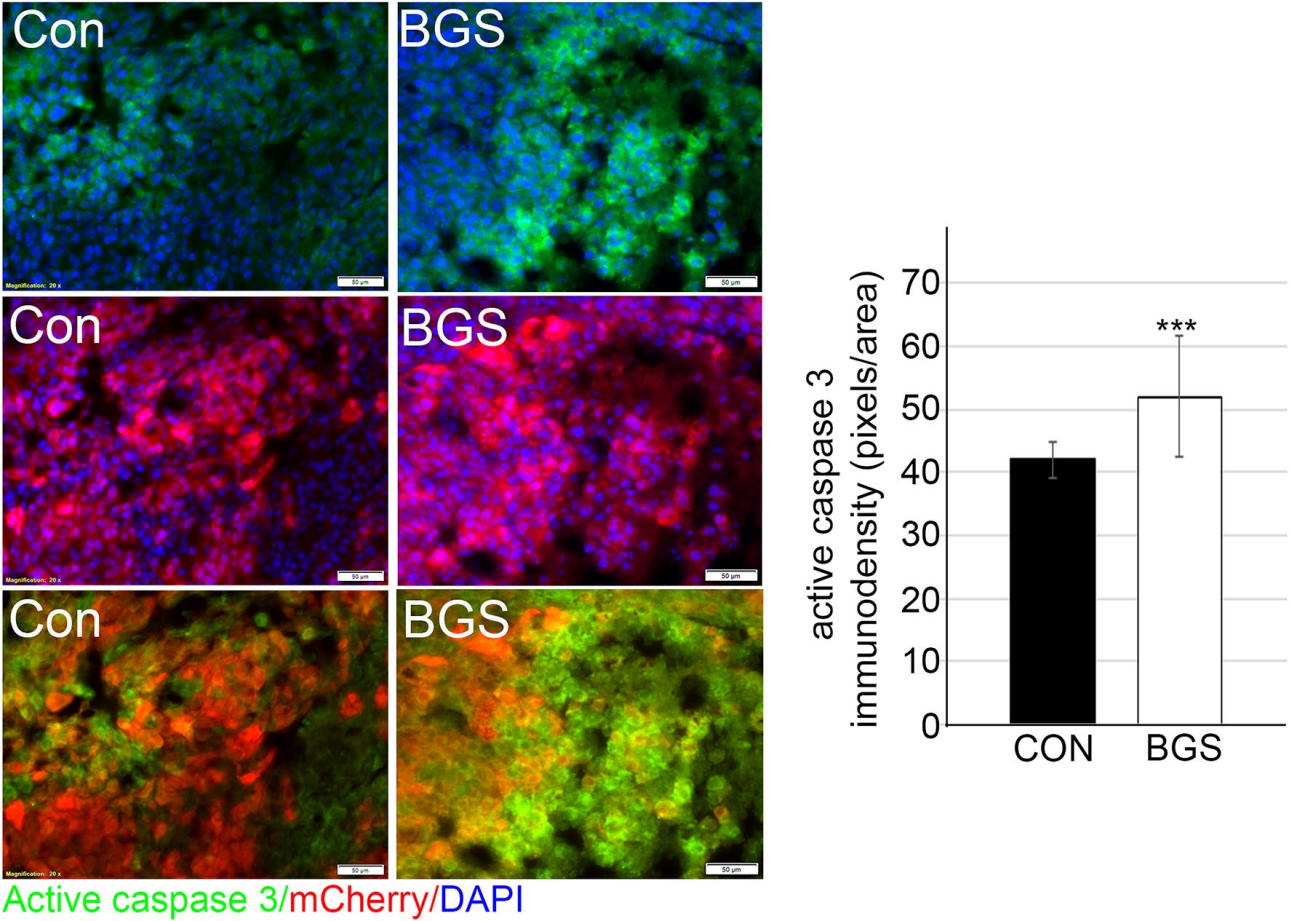
BGS treatment increases RET cell apoptosis. Representative images of the control and BGS treatments for active caspase-3 apoptotic marker expression (green) in the tumor (mCherry). Active caspase-3 density is significantly increased in the BGS-treated group compared to the control group. Results are presented as mean ± SD (n = 7 animals/group; ***p < 0.001). Scale bar 50 µm.

It has been previously demonstrated that DNA strand breaks lead to activation of the apoptotic process, which in turn is regulated by p53 activation and increased caspase-3 expression^45^. The expression of caspase-3 in gastric cancer patients, for example, correlates with better prognosis, and therefore may act as a tumor suppressor^46^. Our examination of active caspase-3 expression revealed that BGS treatment significantly increases caspase-3 expression in RET melanoma cells in vivo (control 42.0 ± 2.89; BGS 52.0 ± 9.6) (Fig. 5). This suggests that BGS treatment facilitates cell death in melanoma cells as a response to DNA damage by depleting brain extracellular Glu.

### BGS treatment mediated CD8^+^ cell recruitment into the tumor and induced p-STAT1 signaling

**Figure 6:**
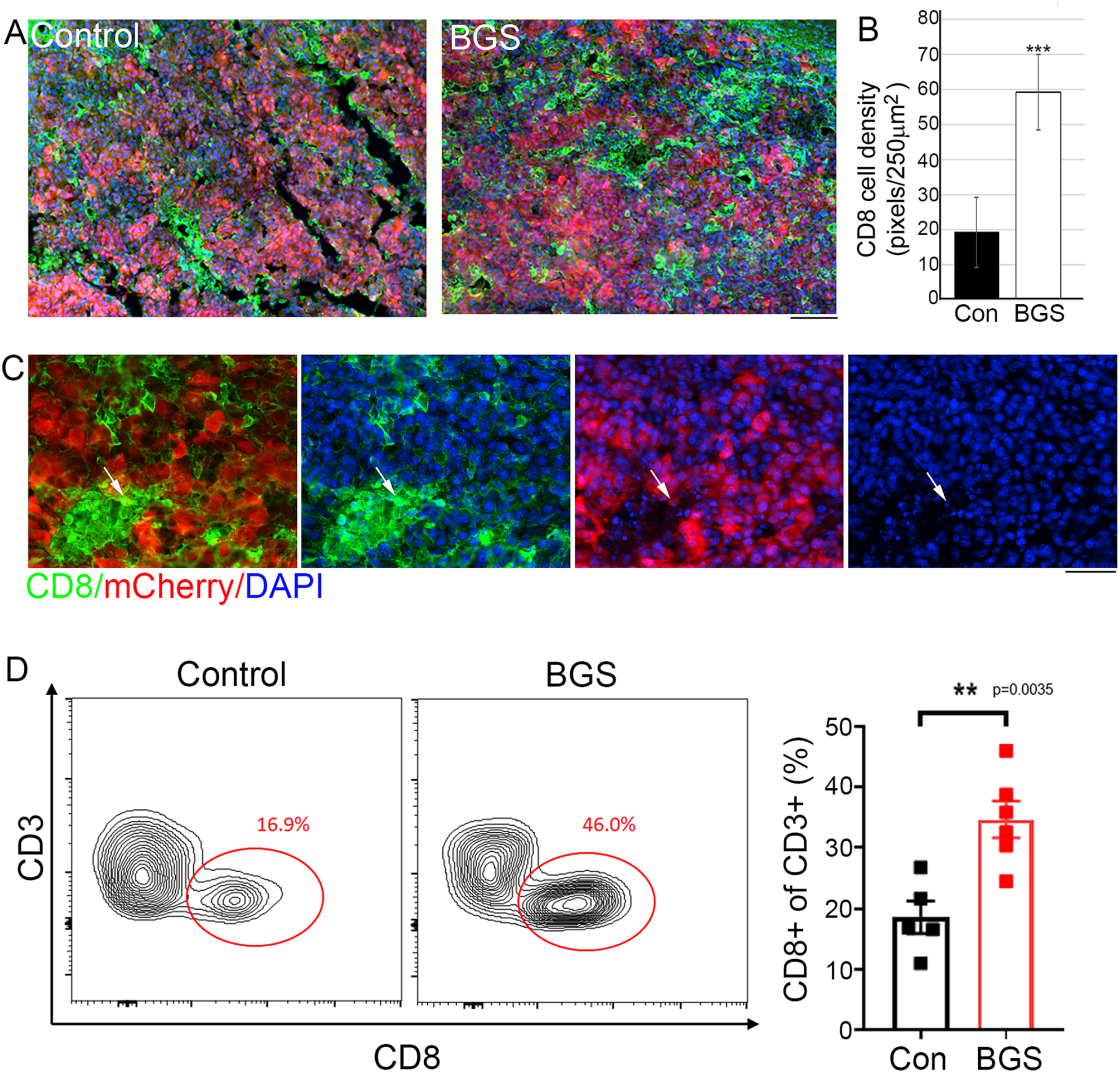
BGS treatment increased CD8 ^+^ cells invasion into the tumor. (**A**) Representative images of the control and BGS treatments for CD8^+^ cells (green) in the tumor (mCherry). Scale bar 200 µm. (**B**) CD8^+^ cell density is significantly increased in the BGS-treated group compared to the control group. Results are presented as mean ± SD (n = 7 animals/group; ***p < 0.001). (**C**) Arrows indicate an example of a CD8^+^ cell phagocyte mCherry positive RET melanoma cell, scale bar 50 µm. (**D**) Flow cytometry analysis of CD3^+^CD8^+^cell frequency in the tumours. Results are presented as mean ± SD (n = 5 brain tumors/each group; **p < 0.0035) from two independent experiments.

**Figure 7:**
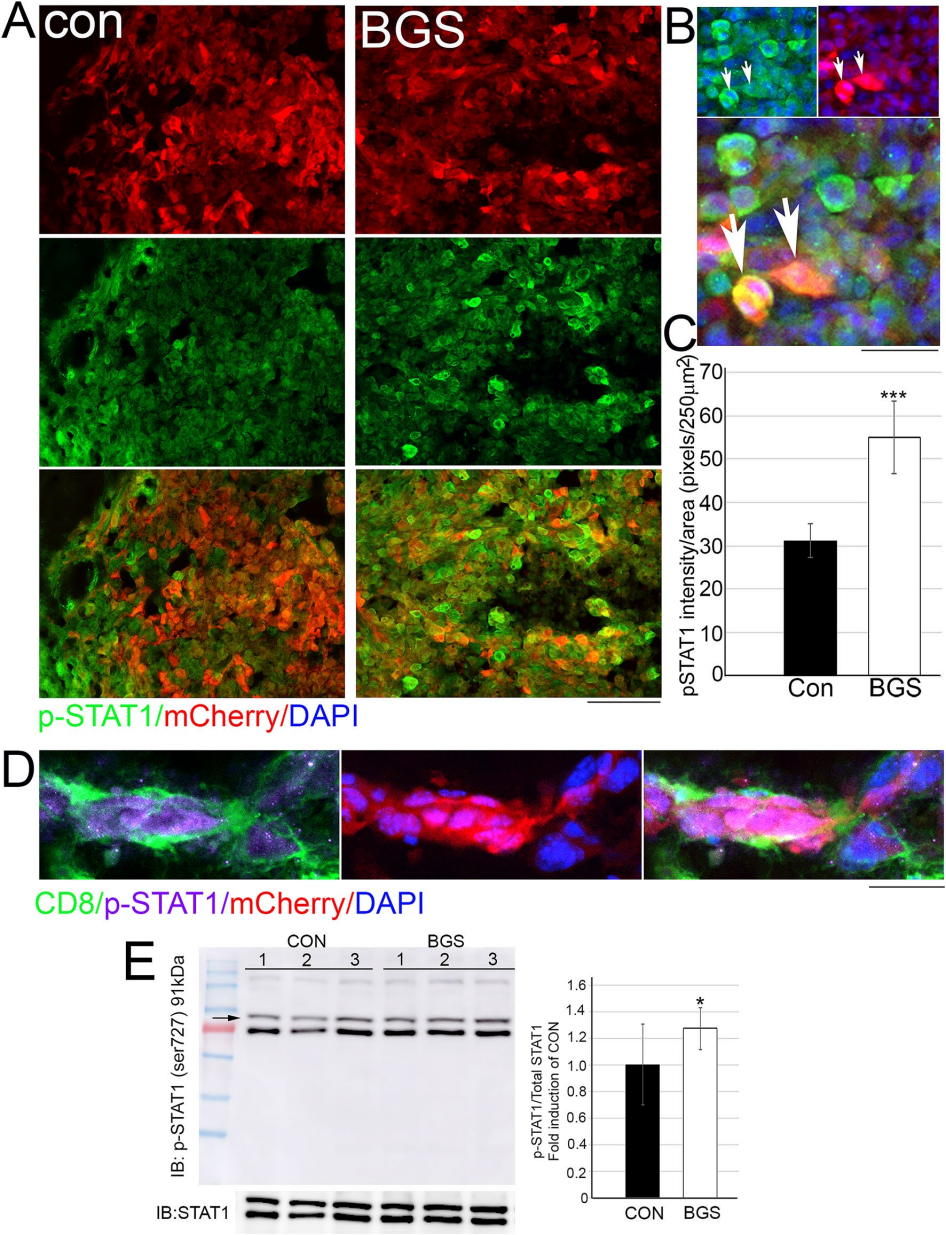
BGS treatment increased p-STAT1 levels in the RET melanoma cells surrounded by CD8^+^ cells. Seven days after RET cell injection (**A**) Representative images of the control and BGS treatments for p-STAT1 signaling in the tumor cells (mCherry), scale bar 200 µm. (**B**) High power magnification of co-localization of p-STAT1 (green) and mCherry RET melanoma cell (red), scale bar 25 µm. (**C**) p-STAT1 density is significantly increased in the BGS-treated group compared to the control group. Results are presented as mean ± SD (n = 7 animals/group; ***p < 0.001). (**D**) An example of a CD8^+^ cell (green) surrounding a mCherry positive RET melanoma cell (red) demonstrated up-regulated p-STAT1 (purple), scale bar 25 µm. (**E**) Tumour lysates from (n = 5 animals/group) were immunoblotted with p-STAT1 (s727) and total anti-STAT1 antibody in order to determine the fold induction of p-STAT1 levels in BGS treated compared to vehicle-control. Results are presented as mean ± SD (*p < 0.05).

Tumor-infiltrating immune cells are associated with tumor prognosis, and a high density of CD8^+^ T cells is associated with a good clinical outcome in various tumor types^47–49^. Since BGS treatment mediated RET melanoma cell death, we further examined whether tumor cell death was accompanied by an invasion of CD8^+^ cytotoxic cells into the tumor area. As demonstrated by both immunohistochemistry and flow cytometry, BGS treatment increased the infiltration of CD8^+^ cells into the tumor (Fig. 6A–C). Flow cytometry analysis revealed 37.4 ± 3.01% of CD8^+^ cells infiltration in BGS treatment, compared to only 18.6 ± 2.65% in the control group (Fig. 6D). The immune response consists of immune cell infiltration into tumor tissue and inhibitory signaling of tumor progression, depending on the tumor microenvironment^50–52^. Therefore, we next assessed the expression of a phosphorylated signal transducer and activator of transcription 1 (p-STAT1), one of the IFN-gamma signaling pathway molecules. Our results demonstrated that the density of p-STAT1 within tumor cells was significantly elevated in the BGS treatment group, when compared with the control group (control 31.13 ± 3.9; BGS 55.12 ± 8.4) (Fig. 7A–C). An increase in p-STAT1 in BGS-treated animals was also confirmed by Western blot analysis (Fig. 7E), with a 27% increase compared to control. Furthermore, RET melanoma cells surrounded by CD8^+^ cells expressed a high level of p-STAT1, which was significantly increased within melanoma cells in the BGS-treated group (Fig. 7D). This strongly supports the theory that BGS treatment mediates CD8^+^ cell infiltration into the tumor and is associated with signaling induction of p-STAT1. This also coincides with signs of tissue damage, such as cell apoptosis.

### BGS treatment mediates phagocytic CD68^+^ macrophages invasion into the tumour

**Figure 8:**
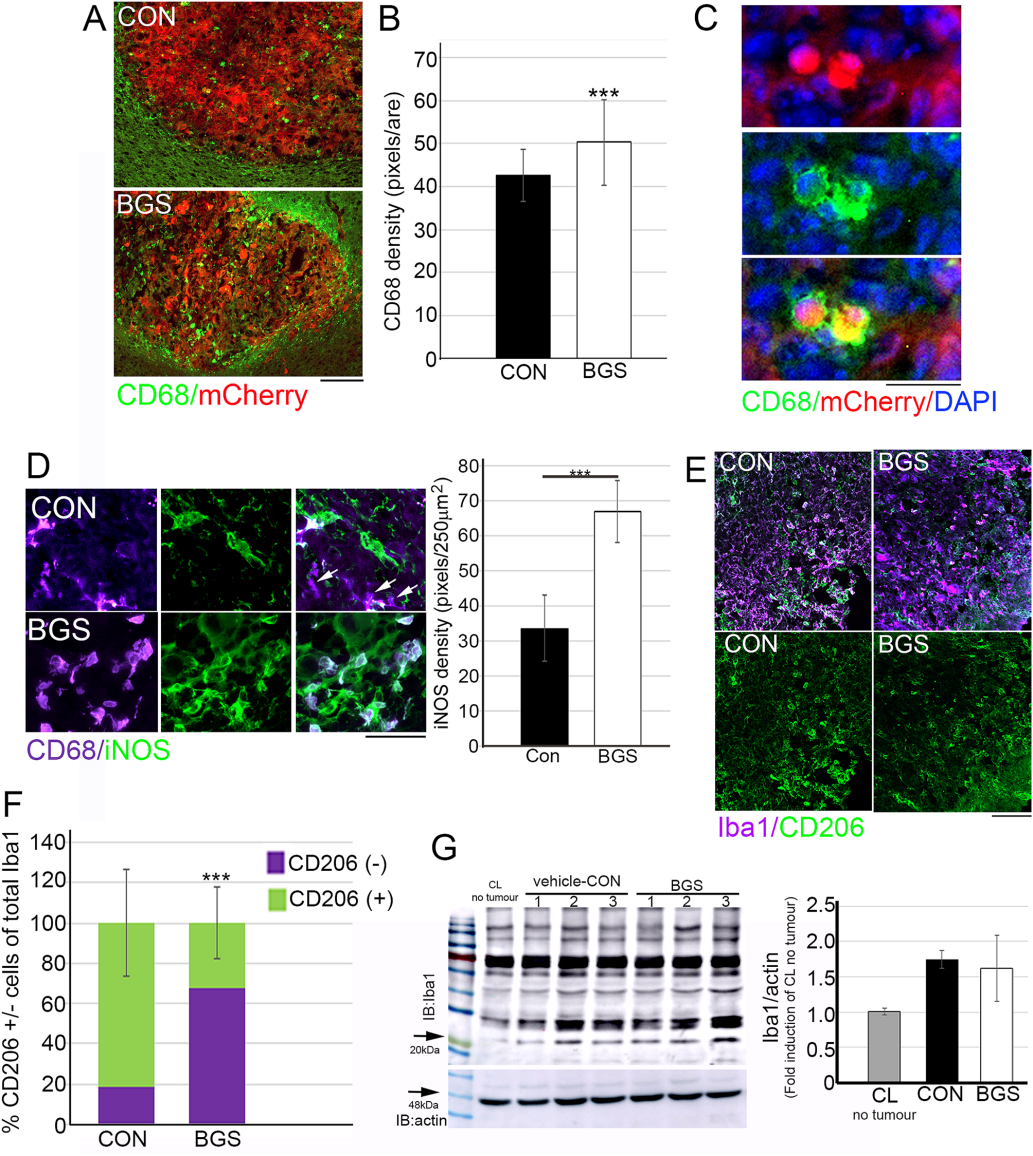
BGS treatment increased CD68^+^ macrophage intratumor invasion and iNOS recreation. (**A**) Representative images of the control and BGS treatments for CD68^+^ macrophage invasion into the tumor (mCherry), scale bar 200 µm. (**B**) CD68^+^ cell density is significantly increased in the BGS-treated group compared to the control group. Results are presented as mean ± STDEV (n = 7 animals/group; ***p < 0.001). (**C**) An example of CD68^+^ cell phagocyte mCherry positive RET melanoma cells, scale bar 25 µm. (**D**) Double immunostaining of CD68^+^ with iNOS demonstrated that in the BGS treatment group most CD68^+^ cells were co-labeled with iNOS as opposed to the control group where significantly less iNOS expression was detected on the CD68^+^ cells and overall. Results are presented as mean ± SD (n = 7 animals/group; ***p < 0.001). Scale bar 50 µm. (**E**) Double immunostaining of CD206 and IBA-1 demonstrated that in the BGS treated group significantly lower number of CD206^+^IBA1^+^ cells as opposed to the control group where significantly higher number CD206 expression was detected on the Iba1 expressing cells. (**F**) Results are presented as mean ± SD (n = 5 animals/group; ***p < 0.001). Scale bar 100 µm. (**G**) Tumour lysates from (n = 5 animals/group) were immunoblotted with Iba1 and actin Abs in order to demonstrated that Iba1 levels were no different between BGS treated compared to vehicle-control. Results are presented as mean ± SD.

We used CD68 as a marker of phagocytic activity of macrophages. An increased invasion of macrophages into the tumor area was observed in the BGS treated mice (Fig. 8A,B). Moreover, RET melanoma cells were detected inside macrophages, as was demonstrated by co-localization of CD68 macrophage and mCherry RET cells with fragmented condensed nuclei (Fig. 8C). These macrophages were co-localized with iNOS immunostaining, suggesting that they are of the pro-inflammatory type of macrophage. In the control group, iNOS density in the tumor area was significantly lower compared to the BGS treatment group (control 33.6 ± 9.4; BGS 67.1 ± 8.8), and many of the CD68 cells were iNOS negative (Fig. 8D; white arrows). This suggests that BGS treatment mediates a pro-inflammatory macrophage phenotype that contributes to the tumor suppression.

To further test this theory, we examined the percentage of cells positive for CD206, a marker of the M2 anti-inflammatory macrophages/microglia type, from the total cells expressing Iba1. Our results demonstrated that the total Iba1 immunofluorescence density was not different in the control vs. BGS groups (control 38.4 ± 9.3; BGS 29.8 ± 14.6), and this was also confirmed by Western blot analysis (Fig. 8G). However, quantitation of CD206^+^ fluorescence out of total Iba1 demonstrated that BGS treatment significantly decreased CD206^+^ cells to 32% compared to 81% in control treatment (Fig. 8E,F). These results strongly suggest that BGS treatment enhances the pro-inflammatory response by the macrophages that invade and surround the tumor.

## Discussion

The blood-glutamate scavenging approach was recently found to be beneficial in attenuating the progression of glioblastomas by reducing excess brain Glu concentrations^5^. Supplementing rGOT1 and its co-substrate, oxaloacetic acid, causes a rapid decrease in blood levels of Glu^36^. In turn, this leads to a larger Glu gradient between the brain and the blood, promoting an efflux of the excess extracellular Glu into the blood^35^. In this study, we examined the anti-tumor effects of such a BGS treatment in a brain metastatic melanoma mice model. The main findings of this study were: (1) Glu concentrations in the CSF consistently increased along with tumor growth, and was significantly higher in implanted animals as compared to healthy mice; (2) The BGS treatment significantly decreased plasma and CSF Glu concentrations; (3) The BGS treatment reduced melanoma growth in vivo by decreasing tumor cell proliferation and by increasing pro-apoptotic signaling; (4) The BGS treatment mediated CD8^+^ T cell recruitment into the tumor; and (5) The BGS treatment enhanced the pro-inflammatory response by decreasing CD206 positive cells, and mediated phagocytic CD68^+^ macrophage invasion into the tumor.

Our results support previous reports that showed an increase in extracellular brain Glu levels in progressed tumors in patients^53,54^. Cancer cells reorganize the body’s metabolism in order to maintain their high proliferation rates. Along with glycolysis and glutaminolysis, amino acids such as Glu are used as an alternative energy source in order to support the biosynthesis of fatty acids. These fatty acids represent part of the secretome of transformed cells, including melanomas^55,56^. Downregulation or blocking of mGluR1 receptors has been shown to result in a decreased rate of metastatic melanoma growth in mice^8,10,12,57,58^. The melanoma lines that were examined in this study showed increased mGluR1 expression, and may have contributed to the high proliferative rate of these cells^11^. Elevation in Glu levels that surround brain tumors may occur due to the increased Glu release by the tumor cells, and/or by neuronal cell death. Decreased Glu uptake caused by an alteration in the expression and function of glutamate excitatory amino acid transporter 2 (EAAT-2) in astrocytes and tumor cells may also contribute to this elevation^59–62^. Tumor cells release Glu mainly via the xc− antiporter, a transporter that plays a critical role in maintaining intracellular and extracellular antioxidant levels (e.g., glutathione, cysteine) by exchanging extracellular cystine for intracellular glutamate^6,63^. Excess extracellular Glu has been shown to enhance glioma and melanoma tumor cell growth and invasion^1,63–66^, and studies have demonstrated that inhibiting the release of Glu results in a decrease of melanoma tumor growth in mGlu1 receptor-expressing melanomas^63,67^. Similarly, our results demonstrate that reducing extracellular Glu levels following BGS treatment inhibits tumor cell proliferation and induces apoptotic tumor cell death. This was manifested by a significant decrease in tumor size and a high expression of apoptotic markers. Increased tumor cell proliferation is a hallmark of malignancy, and Ki67 has been the most widely used proliferation marker in many types of tumors, including melanomas^40,41^. Our results demonstrate that Ki67 was significantly decreased within the tumor area following BGS treatment, demonstrating a potential clinical significance of this treatment. Although BGS-treated animal tumor volume was not significantly smaller at a more advanced stage (14 days after cell transplantation), this trend was still maintained. These results support our previous study in glioblastomas, in which we demonstrated a weaker inhibition of tumor growth by BGS treatment alone vs. a combined treatment approach of BGS with temozolomide^38^, indicating that BGS treatment may be more potent if given in combination with cytotoxic or immunotherapy treatments.

It has been reported that Glu at low physiological concentrations activates human T cells, adhesion, migration, proliferation, intracellular Ca^2^ and more via its GluRs^30,31^. Yet, Glu’s direct effects on T cells depend on its concentration, and may even be inhibitory at excess pathological concentrations^29,31^. The effects of Glu on T cells also depend on the specific GluR types expressed on the target T cell, the T cell’s type and subtype, the T cell’s resting or activated state, and the presence or absence of other concurrent stimuli besides for Glu^68^. Interestingly, while Glu has been shown to suppress the proliferation of activated T cells, it does not affect the proliferation of normal naive/resting human T cells^69,70^. In this study, increased invasion of CD8^+^ cells was observed within the tumor area in BGS treated mice. In addition, melanoma cells surrounded by CD8^+^ cells displayed augmented p-STAT1 signaling, which may be due to IFN-γ secretion by CD8^+^ cells within the tumor microenvironment^71–73^. The tumor-suppressive role of STAT1 has been previously demonstrated, whereby reconstitution of STAT1 in STAT1-deficient murine fibrosarcoma cells significantly suppressed tumorigenicity and metastasis in nude mice^74^. Moreover, a high (vs. low) expression of STAT1 is reported to be associated with a good prognosis in melanoma patients^75^. The binding of IFN-γ to its related receptor leads to the phosphorylation-dependent activation of JAK1/2 and STAT1. p-STAT1 then forms homodimers that translocate to the nucleus and activate the transcription of IFN-γ-stimulated genes^71^. IFN-induced STAT1 can subsequently activate chemokines, such as CXCL9, CXCL10, and CXCL11, which can recruit more CD8^+^ T cells to provide anti-tumor immunity^75,76^. Moreover, STAT1 as a transcription factor induces the transcription of cell cycle and apoptosis-related genes. For instance, STAT1 can arrest the cell cycle in response to IFNγ through direct interactions with cyclin D1 and CDK4 proteins^77^. IFNγ/STAT1-induced expression of caspases 1 and 8 has been reported in cancer cell lines, where STAT1 regulates Bcl-2 family members, including Bcl-xl, Bax, and Bak^78–80^. It has been demonstrated that patients with upregulated CD8 T effector and IFN-γ gene signatures may benefit from immunotherapy^81^.

In addition, it was proposed that a high density of tumor associated macrophages (TAMs) correlates with brain tumor grade, suggesting a supportive role of TAMs in tumor progression^82,83^. Furthermore, it was reported that the M1 macrophage subtype has an inhibitory effect on tumor growth, whereas the M2 subtype plays a supportive role in promoting tumor progression by secreting cell growth cytokines and survival factors^84–86^. In this study, BGS did not alter the total amount of TAM, however, the density of CD68^+^ cells at the tumor border increased in treated mice while the percentage of CD206^+^ from the total amount of macrophages decreased significantly. This indicates conversion of macrophage phenotype from M2 to M1. He et al. showed that the antibiotic doxycycline, reported as a potent anti-cancer treatment in many in vivo models, was able to inhibit macrophage polarization towards the M2 phenotype^87^. Consequently, our results demonstrated a potential role of rGOT1/OxAc treatment in altering the immune-microenvironment in brain metastatic growth, suggesting that it may be a promising new approach to metastatic cancer management^88^.

Not only does Glu facilitate the progression and invasion of tumor cells, but it also plays a main role in the promotion of tumor-associated seizures (TAS)^89^. Seizures occur in about 50–90% of glioma patients^90^. Increased peri-tumoral Glu concentration, as a result of reduced EAAT2 expression and increased system xc− expression has been shown to correlate with the presence of TAS in glioma patients^62,91^. Based on the above studies and the current results, we can assume that BGS treatment, administered shortly after the surgical removal or ablation of a tumor, or in the early stage of the primary or metastatic disease, has a potential to delay tumor recurrence and decrease the probability of TAS development.

Unfortunately, malignancy of the central nervous system has defied all current therapeutic approaches, and an improved understanding of the biology of brain tumors along with new therapeutic options are needed to improve patient survival and control disease progression. This is the first time that the BGS approach was tested in brain metastatic melanoma and it may be of high clinical significance for the future treatment of melanoma patients. However, the ability of the treatment significantly inhibits the tumor cells growing at the very early stage, was less effective at the more progressive stage of the tumor. Consequently, BGS may serve as a monotherapy at the early stage of the disease or as a complimentary treatment that potentially increases the effectiveness of other treatments such as immunotherapy and chemotherapy. It is clear that treatment of such a multifactorial disease as metastatic melanoma ought to involve a complex therapy. Therefore, future studies should focus on evaluating the efficacy of BGS treatment in combination with Temozolomide and check-point immunotherapy (Supplementary Information).

## Supplementary Information

Supplementary Information.

## References

[1] Takano T (2001). Glutamate release promotes growth of malignant gliomas. Nat. Med..

[2] Sontheimer H (2003). Malignant gliomas: Perverting glutamate and ion homeostasis for selective advantage. Trends Neurosci..

[3] Robert SM, Sontheimer H (2014). Glutamate transporters in the biology of malignant gliomas. Cell Mol. Life Sci..

[4] de Groot J, Sontheimer H (2011). Glutamate and the biology of gliomas. Glia.

[5] Ruban A, Berkutzki T, Cooper I, Mohar B, Teichberg VI (2012). Blood glutamate scavengers prolong the survival of rats and mice with brain-implanted gliomas. Investig. New Drugs.

[6] Sharma MK, Seidlitz EP, Singh G (2010). Cancer cells release glutamate via the cystine/glutamate antiporter. Biochem. Biophys. Res. Commun..

[7] Koppula P, Zhang Y, Shi J, Li W, Gan B (2017). The glutamate/cystine antiporter SLC7A11/xCT enhances cancer cell dependency on glucose by exporting glutamate. J. Biol. Chem..

[8] Gelb T (2015). Atypical signaling of metabotropic glutamate receptor 1 in human melanoma cells. Biochem. Pharmacol..

[9] Wall BA (2014). Disruption of GRM1-mediated signalling using riluzole results in DNA damage in melanoma cells. Pigment Cell Melanoma Res.

[10] Wangari-Talbot J, Wall BA, Goydos JS, Chen S (2012). Functional effects of GRM1 suppression in human melanoma cells. Mol. Cancer Res..

[11] Namkoong J (2007). Metabotropic glutamate receptor 1 and glutamate signaling in human melanoma. Cancer Res..

[12] Ohtani Y (2008). Metabotropic glutamate receptor subtype-1 is essential for in vivo growth of melanoma. Oncogene.

[13] Shah R, Singh SJ, Eddy K, Filipp FV, Chen S (2019). Concurrent targeting of glutaminolysis and metabotropic glutamate receptor 1 (GRM1) reduces glutamate bioavailability in GRM1(+) melanoma. Cancer Res..

[14] Pollock PM (2003). Melanoma mouse model implicates metabotropic glutamate signaling in melanocytic neoplasia. Nat. Genet..

[15] Prickett TD, Samuels Y (2012). Molecular pathways: Dysregulated glutamatergic signaling pathways in cancer. Clin. Cancer Res..

[16] Fidler IJ (2003). The pathogenesis of cancer metastasis: The 'seed and soil' hypothesis revisited. Nat. Rev. Cancer.

[17] Sandru A, Voinea S, Panaitescu E, Blidaru A (2014). Survival rates of patients with metastatic malignant melanoma. J. Med. Life.

[18] Vosoughi E (2018). Survival and clinical outcomes of patients with melanoma brain metastasis in the era of checkpoint inhibitors and targeted therapies. BMC Cancer.

[19] Brocke KS (2010). Glutamate receptors in pediatric tumors of the central nervous system. Cancer Biol. Ther..

[20] Koochekpour S (2013). Glutamate, a metabolic biomarker of aggressiveness and a potential therapeutic target for prostate cancer. Asian J. Androl..

[21] Yu LJ, Wall BA, Wangari-Talbot J, Chen S (2017). Metabotropic glutamate receptors in cancer. Neuropharmacology.

[22] Weber J (2011). Immunotherapy for melanoma. Curr. Opin. Oncol..

[23] Wilgenhof S (2011). Restoration of tumor equilibrium after immunotherapy for advanced melanoma: Three illustrative cases. Melanoma Res..

[24] Fonti R, Pellegrino S, Mainolfi CG, Matano E, Del Vecchio S (2020). Brain metastases unresponsive to immunotherapy detected by 18F-FDG-PET/CT in a patient with melanoma. Diagnostics.

[25] Le Rhun E (2020). Response assessment and outcome of combining immunotherapy and radiosurgery for brain metastasis from malignant melanoma. ESMO Open.

[26] Zhang J, Endres S, Kobold S (2019). Enhancing tumor T cell infiltration to enable cancer immunotherapy. Immunotherapy.

[27] Buerki RA, Chheda ZS, Okada H (2018). Immunotherapy of primary brain tumors: Facts and hopes. Clin. Cancer Res..

[28] Taggart D (2018). Anti-PD-1/anti-CTLA-4 efficacy in melanoma brain metastases depends on extracranial disease and augmentation of CD8(+) T cell trafficking. Proc. Natl. Acad. Sci. USA.

[29] Lombardi G (2004). Glutamate modulation of human lymphocyte growth: In vitro studies. Biochem. Biophys. Res. Commun..

[30] Ganor Y, Besser M, Ben-Zakay N, Unger T, Levite M (2003). Human T cells express a functional ionotropic glutamate receptor GluR3, and glutamate by itself triggers integrin-mediated adhesion to laminin and fibronectin and chemotactic migration. J. Immunol..

[31] Ganor Y, Levite M (2014). The neurotransmitter glutamate and human T cells: Glutamate receptors and glutamate-induced direct and potent effects on normal human T cells, cancerous human leukemia and lymphoma T cells, and autoimmune human T cells. J. Neural Transm..

[32] Gottlieb M, Wang Y, Teichberg VI (2003). Blood-mediated scavenging of cerebrospinal fluid glutamate. J. Neurochem..

[33] Cohen-Kashi-Malina K, Cooper I, Teichberg VI (2012). Mechanisms of glutamate efflux at the blood–brain barrier: Involvement of glial cells. J. Cereb. Blood Flow Metab..

[34] Teichberg VI, Cohen-Kashi-Malina K, Cooper I, Zlotnik A (2009). Homeostasis of glutamate in brain fluids: An accelerated brain-to-blood efflux of excess glutamate is produced by blood glutamate scavenging and offers protection from neuropathologies. Neuroscience.

[35] Ruban A, Biton IE, Markovich A, Mirelman D (2015). MRS of brain metabolite levels demonstrates the ability of scavenging of excess brain glutamate to protect against nerve agent induced seizures. Int. J. Mol. Sci..

[36] Perez-Mato M (2014). Human recombinant glutamate oxaloacetate transaminase 1 (GOT1) supplemented with oxaloacetate induces a protective effect after cerebral ischemia. Cell Death Dis..

[37] Goldshmit Y, Banyas E, Bens N, Yakovchuk A, Ruban A (2020). Blood glutamate scavengers and exercises as an effective neuroprotective treatment in mice with spinal cord injury. J. Neurosurg. Spine.

[38] Ruban A, Berkutzki T, Cooper I, Mohar B, Teichberg VI (2012). Blood glutamate scavengers prolong the survival of rats and mice with brain-implanted gliomas. InvestIG. New Drugs.

[39] Goldshmit Y (2018). Blood glutamate scavenger as a novel neuroprotective treatment in spinal cord injury. J. Neurotrauma.

[40] Jovanovic B (2017). A randomized phase II neoadjuvant study of cisplatin, paclitaxel with or without everolimus in patients with stage II/III triple-negative breast cancer (TNBC): Responses and long-term outcome correlated with increased frequency of DNA damage response gene mutations, TNBC subtype, AR status, and Ki67. Clin. Cancer Res..

[41] Kayaselcuk F, Zorludemir S, Gumurduhu D, Zeren H, Erman T (2002). PCNA and Ki-67 in central nervous system tumors: Correlation with the histological type and grade. J. Neurooncol..

[42] Rappold I, Iwabuchi K, Date T, Chen J (2001). Tumor suppressor p53 binding protein 1 (53BP1) is involved in DNA damage-signaling pathways. J. Cell Biol..

[43] Schultz LB, Chehab NH, Malikzay A, Halazonetis TD (2000). p53 binding protein 1 (53BP1) is an early participant in the cellular response to DNA double-strand breaks. J. Cell Biol..

[44] Ward IM, Minn K, van Deursen J, Chen J (2003). p53 Binding protein 53BP1 is required for DNA damage responses and tumor suppression in mice. Mol. Cell Biol..

[45] Manakova S, Puttonen KA, Raasmaja A, Mannisto PT (2003). Ara-C induces apoptosis in monkey fibroblast cells. Toxicol. In Vitro.

[46] Huang KH (2018). Caspase-3, a key apoptotic protein, as a prognostic marker in gastric cancer after curative surgery. Int. J. Surg..

[47] Fridman WH, Pages F, Sautes-Fridman C, Galon J (2012). The immune contexture in human tumours: Impact on clinical outcome. Nat. Rev. Cancer.

[48] Kawai O (2008). Predominant infiltration of macrophages and CD8(+) T Cells in cancer nests is a significant predictor of survival in stage IV nonsmall cell lung cancer. Cancer.

[49] Maimela NR, Liu S, Zhang Y (2019). Fates of CD8+ T cells in tumor microenvironment. Comput. Struct. Biotechnol. J..

[50] Dong J (2016). Prognostic potential of an immune score based on the density of CD8(+) T cells, CD20(+) B cells, and CD33(+)/p-STAT1(+) double-positive cells and HMGB1 expression within cancer nests in stage IIIA gastric cancer patients. Chin. J. Cancer Res..

[51] Ma H (2019). Interferon-alpha promotes immunosuppression through IFNAR1/STAT1 signalling in head and neck squamous cell carcinoma. Br. J. Cancer.

[52] Nakayama Y (2019). PhosphoSTAT1 expression as a potential biomarker for antiPD1/antiPDL1 immunotherapy for breast cancer. Int. J. Oncol..

[53] Marcus HJ, Carpenter KL, Price SJ, Hutchinson PJ (2010). In vivo assessment of high-grade glioma biochemistry using microdialysis: A study of energy-related molecules, growth factors and cytokines. J. Neurooncol..

[54] Roslin M, Henriksson R, Bergstrom P, Ungerstedt U, Bergenheim AT (2003). Baseline levels of glucose metabolites, glutamate and glycerol in malignant glioma assessed by stereotactic microdialysis. J. Neurooncol..

[55] Ratnikov BI, Scott DA, Osterman AL, Smith JW, Ronai ZA (2017). Metabolic rewiring in melanoma. Oncogene.

[56] Filipp FV (2012). Glutamine-fueled mitochondrial metabolism is decoupled from glycolysis in melanoma. Pigment Cell Melanoma Res..

[57] Gelb T (2015). Metabotropic glutamate receptor 1 acts as a dependence receptor creating a requirement for glutamate to sustain the viability and growth of human melanomas. Oncogene.

[58] Lee HJ (2011). Glutamatergic pathway targeting in melanoma: Single-agent and combinatorial therapies. Clin. Cancer Res..

[59] Ye ZC, Rothstein JD, Sontheimer H (1999). Compromised glutamate transport in human glioma cells: Reduction-mislocalization of sodium-dependent glutamate transporters and enhanced activity of cystine-glutamate exchange. J. Neurosci..

[60] Varini K (2012). Mislocalization of the exitatory amino-acid transporters (EAATs) in human astrocytoma and non-astrocytoma cancer cells: Effect of the cell confluence. J. Biomed. Sci..

[61] Mahmoud S, Gharagozloo M, Simard C, Gris D (2019). Astrocytes maintain glutamate homeostasis in the CNS by controlling the balance between glutamate uptake and release. Cells.

[62] Yuen TI (2012). Glutamate is associated with a higher risk of seizures in patients with gliomas. Neurology.

[63] Nagane M (2018). Sulfasalazine, an inhibitor of the cystine-glutamate antiporter, reduces DNA damage repair and enhances radiosensitivity in murine B16F10 melanoma. PLoS ONE.

[64] Sontheimer H (2008). A role for glutamate in growth and invasion of primary brain tumors. J. Neurochem..

[65] Rzeski W, Ikonomidou C, Turski L (2002). Glutamate antagonists limit tumor growth. Biochem. Pharmacol..

[66] Wasinger C, Hofer A, Spadiut O, Hohenegger M (2018). Amino acid signature in human melanoma cell lines from different disease stages. Sci. Rep..

[67] Le MN (2010). The glutamate release inhibitor Riluzole decreases migration, invasion, and proliferation of melanoma cells. J. Investig. Dermatol..

[68] Ganor Y, Teichberg VI, Levite M (2007). TCR activation eliminates glutamate receptor GluR3 from the cell surface of normal human T cells, via an autocrine/paracrine granzyme B-mediated proteolytic cleavage. J. Immunol..

[69] Pacheco R, Gallart T, Lluis C, Franco R (2007). Role of glutamate on T-cell mediated immunity. J. Neuroimmunol..

[70] Pacheco R (2004). Group I metabotropic glutamate receptors mediate a dual role of glutamate in T cell activation. J. Biol. Chem..

[71] Khodarev NN, Roizman B, Weichselbaum RR (2012). Molecular pathways: Interferon/stat1 pathway: Role in the tumor resistance to genotoxic stress and aggressive growth. Clin. Cancer Res..

[72] Goodman ML (2019). Progesterone receptor attenuates STAT1-mediated IFN signaling in breast cancer. J. Immunol..

[73] Owen KL, Brockwell NK, Parker BS (2019). JAK-STAT signaling: A double-edged sword of immune regulation and cancer progression. Cancers.

[74] Huang S, Bucana CD, Van Arsdall M, Fidler IJ (2002). Stat1 negatively regulates angiogenesis, tumorigenicity and metastasis of tumor cells. Oncogene.

[75] Carretero R (2012). Regression of melanoma metastases after immunotherapy is associated with activation of antigen presentation and interferon-mediated rejection genes. Int. J. Cancer.

[76] Au KK (2016). STAT1-associated intratumoural TH1 immunity predicts chemotherapy resistance in high-grade serous ovarian cancer. J. Pathol. Clin. Res..

[77] Dimco G, Knight RA, Latchman DS, Stephanou A (2010). STAT1 interacts directly with cyclin D1/Cdk4 and mediates cell cycle arrest. Cell Cycle.

[78] Chin YE, Kitagawa M, Kuida K, Flavell RA, Fu XY (1997). Activation of the STAT signaling pathway can cause expression of caspase 1 and apoptosis. Mol. Cell Biol..

[79] Ossina NK (1997). Interferon-gamma modulates a p53-independent apoptotic pathway and apoptosis-related gene expression. J. Biol. Chem..

[80] Baratin M (2001). Regression of primary hepatocarcinoma in cancer-prone transgenic mice by local interferon-gamma delivery is associated with macrophages recruitment and nitric oxide production. Cancer Gene Ther..

[81] Kikuchi T (2019). A subset of patients with MSS/MSI-low-colorectal cancer showed increased CD8(+) TILs together with up-regulated IFN-gamma. Oncol. Lett..

[82] Zhou W (2015). Periostin secreted by glioblastoma stem cells recruits M2 tumour-associated macrophages and promotes malignant growth. Nat. Cell Biol..

[83] Mignogna C (2016). A reappraisal of macrophage polarization in glioblastoma: Histopathological and immunohistochemical findings and review of the literature. Pathol. Res. Pract..

[84] Condeelis J, Pollard JW (2006). Macrophages: Obligate partners for tumor cell migration, invasion, and metastasis. Cell.

[85] Sica A, Schioppa T, Mantovani A, Allavena P (2006). Tumour-associated macrophages are a distinct M2 polarised population promoting tumour progression: Potential targets of anti-cancer therapy. Eur. J. Cancer.

[86] Guadagno E (2018). Role of macrophages in brain tumor growth and progression. Int. J. Mol. Sci..

[87] He L, Marneros AG (2014). Doxycycline inhibits polarization of macrophages to the proangiogenic M2-type and subsequent neovascularization. J. Biol. Chem..

[88] Dehne N, Mora J, Namgaladze D, Weigert A, Brune B (2017). Cancer cell and macrophage cross-talk in the tumor microenvironment. Curr. Opin. Pharmacol..

[89] Neal A (2016). Peritumoural glutamate correlates with post-operative seizures in supratentorial gliomas. J. Neurooncol..

[90] Pallud J (2014). Epileptic seizures in diffuse low-grade gliomas in adults. Brain.

[91] Neal A (2019). Glutamate weighted imaging contrast in gliomas with 7Tesla magnetic resonance imaging. Neuroimage Clin..

