# Supplementary figures and images for: Blood glutamate scavengers increase pro-apoptotic signaling and reduce metastatic melanoma growth in-vivo
Source: Sci Rep. 2021 Jul 19;11:14644. doi: 10.1038/s41598-021-94183-8 (PMC8290021; doi:10.1038/s41598-021-94183-8)

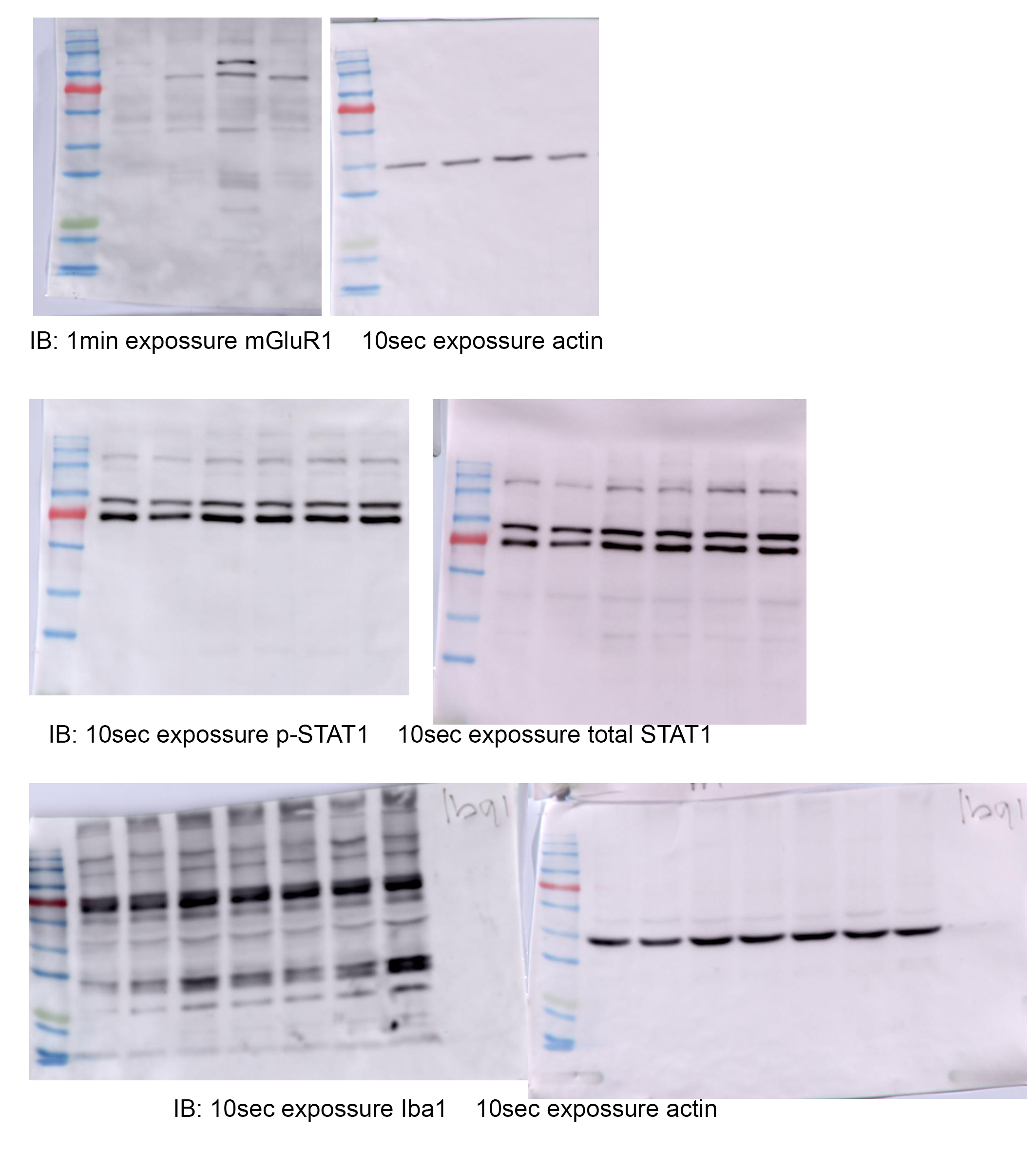

Supplement: Supplementary file 1 — Supplementary Information. [file 41598_2021_94183_MOESM1_ESM.tif]
